# MENTAL HEALTH AND HUNGER: RISK FACTORS FOR HOSPITALIZATION AMONG HOME-DELIVERED MEAL PARTICIPANTS

**DOI:** 10.1093/geroni/igz038.837

**Published:** 2019-11-08

**Authors:** Lisa Juckett

**Affiliations:** The Ohio State University, Columbus, Ohio, United States

## Abstract

Home-delivered meals (HDMs) provided through the Older Americans Act (OAA) are intended to reduce hunger, promote socialization, and maximize wellness. However, HDM recipients are at increased likelihood of being hospitalized due to their complex health needs and risk for social isolation and depression. Drawing data from the OAA-HDM National Survey, we evaluated the predictors of hospitalization among HDM recipients in 2017. From our sample (n = 578), we conducted random forest classification analyses to identify the most important risk factors related to HDM recipient hospitalization. Our random forest model yielded an accuracy rate of 66.3% with risk factors most indicative of hospitalization being attributed to number of co-morbidities, depressive symptoms, and feelings of social isolation. These findings indicate that although HDMs may help alleviate hunger among older adults, innovate strategies are warranted to address the unmet mental health needs of HDM recipients.

